# Supplementary material for: Development of a computer-aided design software for the quantitative evaluation of aesthetic damage
Source: PLoS One. 2019 Dec 18;14(12):e0226322. doi: 10.1371/journal.pone.0226322 (PMC6919621; doi:10.1371/journal.pone.0226322)
Supplement: S1 Code site — 1. (ZIP) [file pone.0226322.s001.zip › [EESC_jr][Projeto]Relatorio_07_21/vendors/select2/docs/_includes/options-old/core-options.html]

# Core options

Select2 supports a small subset of options in every build that is
generated. Each option typically has a decorator that is required that
wraps an adapter, adding support for the option. This is only required
when a custom adapter is being used, as Select2 will build the required
adapters by default.

Select2 will automatically apply decorators to any adapters which have not
been manually overridden. The only time you need to decorate adapters is
when you are using third-party adapters not provided by Select2, or you
are using features not provided in the Select2 core. You can apply a
decorator to an adapter using the
`Utils.Decorate` method provided with
Select2.

```
$.fn.select2.amd.require(
    ["select2/utils", "select2/selection/single", "select2/selection/placeholder"],
    function (Utils, SingleSelection, Placeholder) {
  var CustomSelectionAdapter = Utils.Decorate(SingleSelection, Placeholder);
});
```

All core options that use decorators or adapters will clearly state it
in the "Decorator" or "Adapter" part of the documentation. Decorators are
typically only compatible with a specific type of adapter, so make sure to
note what adapter is given.

## Declaring configuration in the `data-*` attributes

It is recommended that you declare your configuration options for Select2
when initializing Select2. You can also define your configuration options
by using the HTML5 `data-*` attributes, which will override
any options set when initializing Select2 and any defaults.

This means that if you declare your `<select>` tag as...

```
<select data-tags="true" data-placeholder="Select an option"></select>
```

Will be interpreted the same as initializing Select2 as...

```
$("select").select2({
  tags: "true",
  placeholder: "Select an option"
});
```

You can also define nested configurations, which are typically needed for
options such as AJAX. Each level of nesting should be separated by two
dashes (`--`) instead of one. Due to
a jQuery bug,
nested options using `data-*` attributes
do not work in jQuery 1.x.

```
<select data-ajax--url="http://example.org/api/test" data-ajax--cache="true"></select>
```

Which will be interpreted the same as initializing Select2 with...

```
$("select").select2({
  ajax: {
    url: "http://example.org/api/test",
    cache: "true"
  }
});
```

The value of the option is subject to jQuery's
parsing rules for
HTML5 data attributes.

## AMD compatibility

You can find more information on how to integrate Select2 with your
existing AMD-based project by
viewing the 4.0 release notes.
Select2 automatically loads some modules when the adapters are being
automatically constructed, so those who are using Select2 with a custom
AMD build using their own system may need to specify the paths that are
generated to the Select2 modules.

Key
:   `amdBase`

Default
:   `select2/`

Key
:   `amdLanguageBase`

Default
:   `select2/i18n/`

## Displaying selections

Select2 provides options that allow you to directly affect how the
container that holds the current selection is displayed.

### Placeholders

Select2 can display a placeholder for a single-value select that will
replace an option, or be shown when no options are selected for
multiple-value selects. You can find an example on the
example page.

Key
:   `placeholder`

Value
:   string or object

---

Adapter
:   `SelectionAdapter`

Decorator
:   `Placeholder`
    and
    `HidePlaceholder`

**Heads up!**
Because browsers assume that the first `option` in
single-value select boxes is selected, you should add an empty
`<option></option>` tag that the placeholder
should use or it may not work.

If the **value is a string**, the placeholder will be
displayed when a **blank option** is used as the placeholder.
The **value** will be the message to show to users as the
placeholders.

```
placeholder: "Select a repository"
```

If the **value is an object**, the object should be
compatible with Select2's internal objects. The `id` should
be the id to look for when determining if the placeholder should be
displayed. The `text` should be the placeholder to display
when that option is selected.

```
placeholder: {
  id: "-1",
  text: "Select a repository"
}
```

You should **pass in an object** when you are using a
framework that **creates its own placeholder option**. The
**id** should be the same as the `value`
attribute on the `option`.

You can allow a selected option to be cleared back to the placeholder by
enabling the `allowClear` option.

Key
:   `allowClear`

Value
:   boolean

Adapter
:   `SelectionAdapter`

Decorator
:   `AllowClear`

This will display an "x" that the user can click to clear the current
selection. It is designed to be used for cases where a single selection
can be made.

### Multiple selections

Select2 can display either a single selection or multiple selections.

Key
:   `multiple`

Value
:   boolean (`true` or `false`)

This option will determine what the `SelectAdapter` (used by
default) should use to set the value of the underlying `select`
element. It will also determine if the `MultipleSelection`
adapter should be used.

### Container width

Select2 will try to match the width of the original element as closely as
possible. Sometimes this isn't perfect, which is what you can tell Select2
how to determine the width.

| Value | Description |
| --- | --- |
| `"element"` | Uses javascript to calculate the width of the source element. |
| `"style"` | Copies the value of the width `style` attribute set on the source element. |
| `"resolve"` | Tries to use `style` to determine the width, falling back to `element`. |
| Anything else | The value of the `width` option is directly set as the width of the container. |

Key
:   `width`

Value
:   string

### Internationalization (Language support)

Messages will be displayed to users when necessary, such as when no
search results were found or more characters need to be entered in order
for a search to be made. These messages have been
translated into many languages
by contributors to Select2, but you can also provide your own
translations.

Key
:   `language`

Value
:   object or string

---

Module
:   `Translation`

**Heads up!** When using translations provided by Select2,
you must make sure to include the translation file in your page after
Select2.

When a string is passed in as the language, Select2 will try to resolve
it into a language file. This allows you to specify your own language
files, which must be defined as an AMD module. If the language file
cannot be found, Select2 will assume it is a language code controlled by
Select2, and it will try to load the translations for that language
instead.

You can include your own translations by providing an object similar to
the one below.

```
language: {
  // You can find all of the options in the language files provided in the
  // build. They all must be functions that return the string that should be
  // displayed.
  inputTooShort: function () {
    return "You must enter more characters...";
  }
}
```

### Templating results and selections

By default, Select2 will display the option text within the list of
results and when the option has been selected. Select2 comes with options
that allow you to further customize the display of results and selections,
allowing you to display them however you want.

#### Customizing the display of selections

When an option is displayed after it has been selected, it is passed
through a formatting function that determines what is displayed. By
default, the function only returns the `text` key of the data
object.

Key
:   `templateSelection`

Value
:   A function taking a `selection` object

**Anything rendered as a selection is templated.**
This includes placeholders and pre-existing selections that are displayed,
so you must ensure that your templating functions can support them.

The `templateSelection` function should return a string
containing the text to be displayed, or an object (such as a jQuery
object) that contains the data that should be displayed.

**Strings are assumed to contain only text** and will be
passed through the `escapeMarkup` function, which strips any
HTML markup.

**Anything else will be passed
directly to `jQuery.fn.append`**  and will be handled directly by jQuery. Any markup, such as
HTML, returned will not be escaped and it is up to you to escape any
malicious input provided by users.

#### Customizing the display of results

When an option is displayed after it has been selected, it is passed
through a formatting function that determines what is displayed. By
default, the function only returns the `text` key of the data
object.

Key
:   `templateResult`

Value
:   A function taking a `result` object

**Anything rendered in the results is templated.**
This includes results such as the "Searching..." and "Loading more..."
text which will periodically be displayed, which allows you to add more
advanced formatting to these automatically generated options.

The `templateResult` function should return a string
containing the text to be displayed, or an object (such as a jQuery
object) that contains the data that should be displayed. It can also
return `null`, which will prevent the option from being
displayed in the results list.

**Strings are assumed to contain only text** and will be
passed through the `escapeMarkup` function, which strips any
HTML markup.

**Anything else will be passed
directly to `jQuery.fn.append`**  and will be handled directly by jQuery. Any markup, such as
HTML, returned will not be escaped and it is up to you to escape any
malicious input provided by users.

## Returning and displaying results

Select2 can work on many different data sets ranging from local options,
the same way that a `<select>` typically works, from
remote options where a server generates the results that users can select
from.

### Array

Select2 allows creating the results based on an array of data objects that
is included when initializing Select2.

Key
:   `data`

Value
:   array of objects

Adapter
:   `ArrayAdapter`

The objects that the users can select from should be passed as an array
with each object containing `id` and `text`
properties.

### AJAX

Select2 allows searching for results from remote data sources using AJAX
requests.

Key
:   `ajax`

Value
:   object

Adapter
:   `AjaxAdapter`

All options passed to this option will be directly passed to the
`$.ajax` function that executes AJAX requests. There are a few
custom options that Select2 will intercept, allowing you to customize the
request as it is being made.

```
ajax: {
  // The number of milliseconds to wait for the user to stop typing before
  // issuing the ajax request.
  delay: 250,
  // You can craft a custom url based on the parameters that are passed into the
  // request. This is useful if you are using a framework which has
  // JavaScript-based functions for generating the urls to make requests to.
  //
  // @param params The object containing the parameters used to generate the
  //   request.
  // @returns The url that the request should be made to.
  url: function (params) {
    return UrlGenerator.Random();
  },
  // You can pass custom data into the request based on the parameters used to
  // make the request. For `GET` requests, the default method, these are the
  // query parameters that are appended to the url. For `POST` requests, this
  // is the form data that will be passed into the request. For other requests,
  // the data returned from here should be customized based on what jQuery and
  // your server are expecting.
  //
  // @param params The object containing the parameters used to generate the
  //   request.
  // @returns Data to be directly passed into the request.
  data: function (params) {
    var queryParameters = {
      q: params.term
    }

    return queryParameters;
  },
  // You can modify the results that are returned from the server, allowing you
  // to make last-minute changes to the data, or find the correct part of the
  // response to pass to Select2. Keep in mind that results should be passed as
  // an array of objects.
  //
  // @param data The data as it is returned directly by jQuery.
  // @returns An object containing the results data as well as any required
  //   metadata that is used by plugins. The object should contain an array of
  //   data objects as the `results` key.
  processResults: function (data) {
    return {
      results: data
    };
  },
  // You can use a custom AJAX transport function if you do not want to use the
  // default one provided by jQuery.
  //
  // @param params The object containing the parameters used to generate the
  //   request.
  // @param success A callback function that takes `data`, the results from the
  //   request.
  // @param failure A callback function that indicates that the request could
  //   not be completed.
  // @returns An object that has an `abort` function that can be called to abort
  //   the request if needed.
  transport: function (params, success, failure) {
    var $request = $.ajax(params);

    $request.then(success);
    $request.fail(failure);

    return $request;
  }
}
```

### Tags

Users can create their own options based on the text that they have
entered.

Key
:   `tags`

Value
:   boolean / array of objects

Adapter
:   `DataAdapter`

Decorator
:   `Tags`

If the `tags` option is passed into Select2, if a user types
anything into the search box which doesn't already exist, it will be
displayed at the top and the user will be able to select it.

**For backwards compatibility**, if an array of objects is
passed in with the `tags` option, the options will be
automatically created and the user will be able to select from them.
This is the **same as how array data
works**, and has similar limitations.

### Change how options are matched when searching

When users filter down the results by entering search terms into the
search box, Select2 uses an internal "matcher" to match search terms to
results. **When a remote data set is used, Select2 expects that the
returned results have already been filtered.**

Key
:   `matcher`

Value
:   A function taking search `params` and the
    `data` object.

Select2 will pass the individual data objects that have been passed back
from the data adapter into the `matcher` individually to
determine if they should be displayed. Only the first-level objects will
be passed in, so **if you are working with nested data, you need to
match those individually**.

```
matcher: function (params, data) {
  // If there are no search terms, return all of the data
  if ($.trim(params.term) === '') {
    return data;
  }

  // `params.term` should be the term that is used for searching
  // `data.text` is the text that is displayed for the data object
  if (data.text.indexOf(params.term) > -1) {
    var modifiedData = $.extend({}, data, true);
    modifiedData.text += ' (matched)';

    // You can return modified objects from here
    // This includes matching the `children` how you want in nested data sets
    return modifiedData;
  }

  // Return `null` if the term should not be displayed
  return null;
}
```

This allows for more advanced matching when working with nested objects,
allowing you to handle them however you want. For those who are not
looking to implement highly customized matching, but instead are just
looking to change the matching algorithm for the text, a
compatibility modules has been created to
make it easier.
